# Volunteering and Cognitive Health Among Middle-Aged and Older Adults: The Mediating Role of Friendship

**DOI:** 10.1093/geroni/igaa057.1340

**Published:** 2020-12-16

**Authors:** Changmin Peng, Jeffrey Burr, Sae Hwang Han, Kyungmin Kim, Jan Mutchler

**Affiliations:** 1 University of Massachusetts Boston, Boston, Massachusetts, United States; 2 University of Texas at Austin, Austin, Texas, United States

## Abstract

We lack knowledge about the underlying mechanisms that link formal volunteering to cognitive health. Friendships can be formed and improved through volunteering. Friendship is also beneficial to cognitive health as it often involves sharing information and promoting social interactions. This study investigated the potential mediating role of friendship (i.e., the number of close friends and the quality of friendships) for the association between volunteering (i.e., volunteering status and volunteering hours) and episodic memory among middle-aged and older adults in the United States, using data from the 2014 wave of the Health and Retirement Study (N = 6,029). Moderated mediation models were employed to test the mediation role of friendship for the association between volunteering and cognition for two age groups, middle-aged adults (age 50-64, n = 2,441) and older adults (age 65+, n = 3,588). The results showed that the quality of friendships, but not the number of close friends, mediated the relationship between volunteering (both status and hours) and episodic memory for middle-aged adults. However, mediation effects of friendship were not discovered for older adults. Our findings emphasize the important role of the quality of friendship in understanding the cognitive benefit of volunteering among middle-aged adults. For older adults, the nonsignificant effects of friendship may be consistent with socioemotional selectivity theory, suggesting that older adults may not use volunteering to expand their social networks. Instead, they may participate in volunteer work for other reasons in the context of shrinking social networks.

